# Phosphatidylcholine Enhances Homeostasis in Peach Seedling Cell Membrane and Increases Its Salt Stress Tolerance by Phosphatidic Acid

**DOI:** 10.3390/ijms23052585

**Published:** 2022-02-26

**Authors:** Maoxiang Sun, Xiaolong Liu, Huaifeng Gao, Binbin Zhang, Futian Peng, Yuansong Xiao

**Affiliations:** State Key Laboratory of Crop Biology, College of Horticulture Science and Engineering, Shandong Agricultural University, Tai’an 271018, China; maoxiangs0514@163.com (M.S.); lxl17860721016@163.com (X.L.); gaohuaifeng1992@163.com (H.G.); zhangbinbin199212@163.com (B.Z.)

**Keywords:** salt stress, phosphatidylcholine, cell membranes, peach, phosphatidic acid

## Abstract

Salt stress is a major adverse abiotic factor seriously affecting fruit tree growth and development. It ultimately lowers fruit quality and reduces yield. Phosphatidylcholine (PC) is an important cell membrane component that is critical for cell structure and membrane stability maintenance. In this study, we found that the addition of external PC sources significantly increased the tolerance of one-year-old peach trees, *Prunus persica* (L.) Batsch., to salt stress and attenuated their damage. The effect of exogenous application of 200 mg/L PC exerted the most significant positive effect. Its use caused seedling leaf stomatal opening, contributing to normal gas exchange. Moreover, beneficial effects were exerted also to the root system, which grew normally under salt stress. Meanwhile, phospholipase D activity in the cell was promoted. The production of phosphatidic acid (PA) was enhanced by increased decomposition of phospholipids; PA serves as a secondary messenger involved in plant biological process regulation and the reduction in the reactive oxygen species- and peroxide-induced damage caused by salt stress. The possible mechanism of action is via promoted plant osmotic regulation and tolerance to salt stress, reducing salt stress-induced injury to plants.

## 1. Introduction

More than 6% of the world’s total land area (approximately 800 million hectares) is affected by salinity [1]. Poor irrigation techniques, inappropriate fertilizer application, and the excessive accumulation of industrial pollutants have contributed to an increase in soil salinity [2]. Under salt stress, plant chloroplasts are destroyed, which decreases the activity of related photosynthetic enzymes, reducing plant photosynthetic rate [3]. Meanwhile, the accumulation of salt ions diminishes the content of thylakoid membrane glycolipids and unsaturated fatty acids, which affects the photosynthetic characteristics of the cell membrane [3]. Due to the increase in the sodium ion level, plants suffer from oxidative stress and ion damage, which increases the permeability of the cell membrane and disrupts the ion balance in the plant. Protein and cell membrane structures and functions are disrupted, which hinders plant growth and development. Ultimately, a continuous high-salt environment causes plant death, resulting in considerably reduced crop yields [4].

The cell membrane serves as a barrier and interface for physical–chemical and information exchange with their external environment. The stability of its structural integrity and its functions are the basis for normal cell metabolism and overall physiology [5]. Adverse conditions, such as ion poisoning and low-temperature and drought stress, initially and directly attack the plant cell membrane, destroying its structure and reducing its fluidity.

Meanwhile, the lipid composition and content in the cell membrane directly affects the cell membrane structure stability and fluidity [6,7]. Continuous adverse influence disrupts cell membrane integrity, fluidity, and selective permeability, causing loss of basic functions of the entire plant cell [8]. Under adverse stress conditions, plants strive to find new ways to reduce the damage caused through long-term adaptive evolution. One of the most important plant survival mechanisms is changing the content and composition of membrane lipids for adaptation to the action of adverse factors [9].

Phosphatidylcholine (PC) is the most abundant phospholipid of all eukaryotic cell membrane components. It is present in high levels in the photosynthetic and non-photosynthetic organs of Arabidopsis thaliana [10]. In addition being a major phospholipid in the cellular membranes of most eukaryotes [11], PC is an important precursor in lipid signaling or serves as a regulatory protein ligand [12]. 

Phospholipase D (PLD) can hydrolyze phospholipids, and its products are directly used as signal molecules involved in stress response signal transduction [13]. PLD regulates cell physiological processes by governing the spatial distribution, temporal and spatial expression, and the content of its product, phosphatidic acid (PA) [14]. Hong et al. [15] presented data on PLD and PA signaling in response to drought and salinity. PA can be directly produced by PLD hydrolysis of phospholipids. Earlier genetic and pharmacological research found that PA plays an important role in the regulation of stomatal movement, root growth, and plant tolerance to salinity and water stress [16].

Reduced PC content and defective plant growth was recently reported in a double mutant of A. thaliana, phospho-baseN-methyltransferase1 (PMT1) and PMT3 [17]. Additionally, Shimojima et al. established that, under exposure to environmental stress factors, plants reduce the phospholipid content of their cell membranes to maintain their integrity [18]. Plants then replace the missing phospholipids by increasing the content of glycolipids in the cell membrane. With the advances in the technologies for research on membrane lipids, increasingly more scholars have devoted to studying the relationship between the changes in cell membrane lipid content and cell membrane structure homeostasis. However, few reports are available on the associations between the changes in plant PC content and plant tolerance to environmental stress. Therefore, we used peach seedlings as experimental material to examine the effects of exogenous PC on the growth of the root and photosynthetic organs of peach seedlings under salt stress. We also investigated their influence on the membrane lipid content and the composition of the leaves and roots. We aimed to elucidate whether exogenous PC treatment can enhance the activity of PLD, improve the salt tolerance of plants, and reduce the damage caused by salt stress exposure to plants.

## 2. Results

### 2.1. Exogenous Application of PC Improved the Net Photosynthetic Rate and Chlorophyll Content of Peach Leaves

Leaves are the main photosynthetic organs in higher plants. The chloroplast in the leaf consists of three parts: envelope, stroma, and thylakoid, which is the main place where photosynthesis occurs. Phospholipid is an important component of the chloroplast membrane.

**Figure 1:**
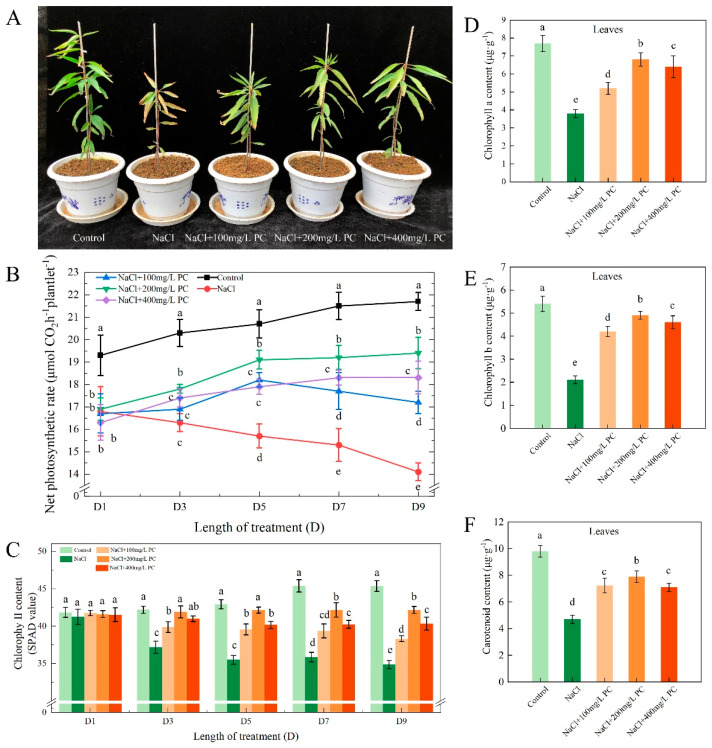
Plant photosynthetic system parameters under salt stress. (**A**) Peach seedlings under salt stress; (**B**) Net photosynthetic rate; (**C**) Chlorophyll content in 1–9 days; (**D**–**F**) Chlorophyll a, b, and carotenoid content on the ninth day. The photos in Figure 1A were taken on the 11th day under salt stress. The error bar represents the standard deviation of the mean (*n* = 3). Different lowercase letters indicate significant differences among treatments (Duncan test, 21.7%, 22.9%; *p* < 0.05).

We found that 200 mg/L PC had a better alleviation effect on NaCl concentration of 70 mmol/L, so NaCl + 200 mg/L PC was selected as one of the treatments. The possible reason is that 200 mg/L PC is the optimum concentration for peach seedlings to resist 70 mmol/L NaCl. As can be observed in Figure 1A, the salt stress treatment not only hindered the development of the peach plants, but also caused leaf yellowing and negatively affected the photosynthetic system of the plant. Our research found that the net photosynthetic rate value in the control treatment increased slightly over time. Compared with the control treatment, the net photosynthetic rate value of the NaCl treatment gradually decreased. The treatments of NaCl + 100 mg/L PC, NaCl + 200 mg/L PC, and NaCl + 400 mg/L PC showed that the net photosynthetic rate was always lower than the control treatment and higher than the NaCl treatment. The net photosynthetic rate in the salt treatments supplemented with 200 mg/L PC and 400 mg/L PC slowly increased from the first to the fifth day and then stabilized from the fifth to the ninth day. The treatment with NaCl + 200 mg/L PC alleviated more significantly the adverse influence of salt stress. Among them, the treatment with NaCl + 200 mg/L PC alleviated more significantly the adverse influence of salt stress. From the third to the ninth day, the net photosynthetic rate of the NaCl + 200 mg/L PC treatment increased by 9.0%, 19.5%, 21.7%, and 22.9% compared with the NaCl treatment (Figure 1B). As can be seen in Figure 1C, salt stress had a greater impact on the chlorophyll content of peach leaves. Salt stress exposure prolongation gradually decreased the chlorophyll content in the leaves. Compared with NaCl treatment, adding PC can significantly reduce the decrease in chlorophyll content. We measured chlorophyll a, chlorophyll b and carotenoids on the ninth day of salt stress, and found that the treatment of NaCl + 200 mg/L PC had higher values than the other treatments. Chlorophyll a, chlorophyll b and carotenoids of the NaCl + 200 mg/L PC treatment increased by 78.9%, 133.3%, and 68.1% compared with the NaCl treatment (Figure 1D–F). 

### 2.2. Effect of PC on Stomatal Density and Size

The stomata are pores through which plants exchange gas with the outside environment; transpiration intensity is also controlled by adaptive changes in their structure. The opening and closing of the stomata are closely related to plant photosynthesis since the CO_2_ needed for photosynthesis can enter the plant organism only when the stomata are open.

**Figure 2:**
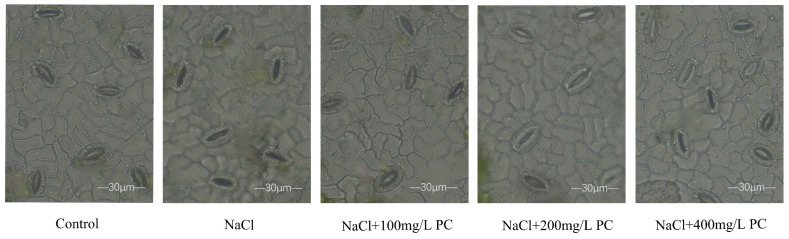
The cell stomatal size of peach tree leaves among different treatments. Measurements were performed using a light microscope to visualize peeled impressions of peach tree leaf epidermis. Peach tree leaf cell stomata were sampled and observed at 10:30 a.m. on the 10th day of the treatment experiment. All samples are measured at approximately the same time of day. Bars correspond to 30 μm.

Our observation under 400x magnification showed that the stomata of the peach leaves in the control treatment were open and the shape of the stomata guard cells was round and full. The NaCl treatment resulted in guard cell closure. On the contrary, the shape of the stomata treated with three salt stress supplemented with PC is relatively full, but there are also stomata in the closed state (Figure 2). Of them, the stomata treated with 200 mg/L PC was in the open state, indicating that the gas normally interacted with the outside environment. The data of the length, width, and area of the stomata are presented in Table 1. As can be seen, the average area of the stomata in the salt treatments was smaller than the untreated control, whereas the area of the stomata in the treatments with PC supplementation was larger. The largest guard cell area was established in the PC-supplemented treatment with 200 mg/L. Compared with the NaCl treatment, the NaCl + 200 mg/L PC treatment had 28.3% larger guard cell width and 37.4% greater area.

### 2.3. Structural Changes in the Peach Root System under Salt Stress

**Figure 3:**
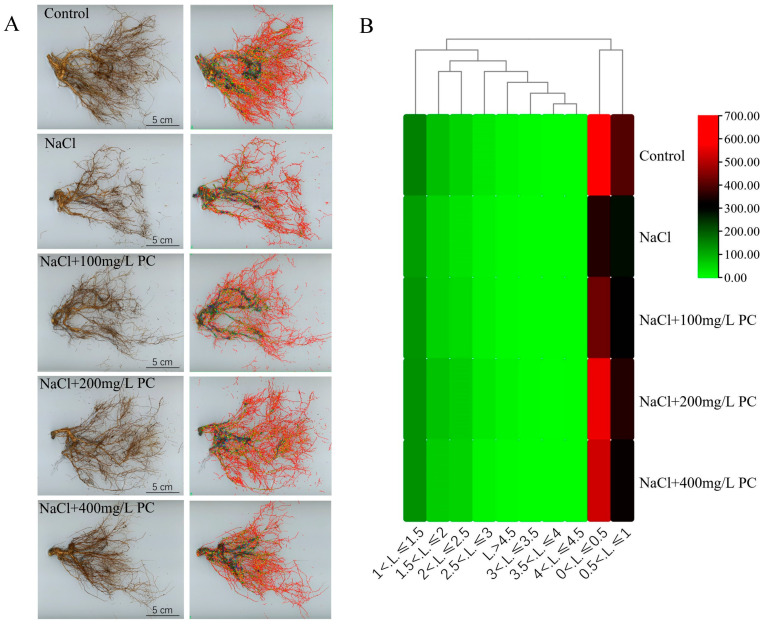
Structural changes in the peach root system under salt stress. (**A**) Photo of the peach tree root structure, the figures of roots in the second column are where the data were collected by the root analysis software. The collected data are root length, surface area, root volume, tips, and forks. (**B**) Fine root distribution heat map. The vast majority of root lengths were distributed in the range of 0 < L ≤ 1, and the root number of NaCl + 200 mg/L PC treatment was the highest in the range of 0 < L ≤ 0.5 under salt stress. Measurements were performed using the intact root system of the peach plant. Measurements of root structural were performed at 10:00 a.m. on 11th day of the salt stress treatment. Bars correspond to 5 cm.

The main functions of plant roots are to fix the aboveground plant parts and absorb water and nutrients from the soil. Root hairs are the main part responsible for the absorption of water and nutrient compounds. Hence, the root hair growth and volume in the soil also determines the potential of plants for nutrient and water absorption. In this research, we found that salt stress considerably reduced the number of absorbing roots (Figure 3A). However, the PC supplementation applied along with the salt stress treatment obviously alleviated the decrease in the number of absorbing roots (Figure 3B). As displayed in Table 2, the salt stress treatment decreased the total length, total surface area, and total volume of the root system. The NaCl + 200 mg/L PC treatment had the greatest positive impact on the attenuation of the reduction in the number of absorbing roots. The total length, total surface area, total volume, and the tips and forks of the root system were 65.23%, 41.01%, 20.36%, 66.99%, and 84.1% higher than those in the NaCl treatment, respectively.

### 2.4. Root Cells and Cell Membrane Integrity of Peach Trees under Salt Stress Exposure

**Figure 4:**
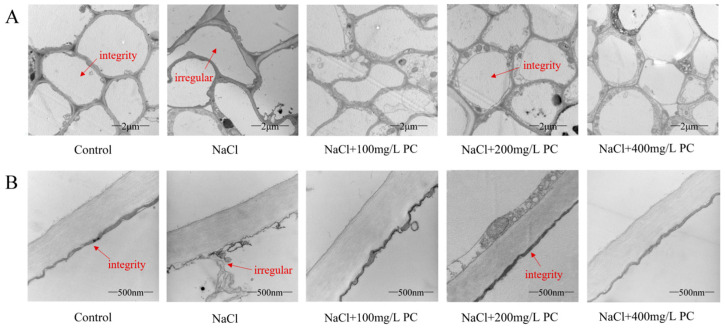
Transmission electron micrograph of peach tree root cells and cell membranes. (**A**) Root cell integrity. Bars correspond to 2 μm; (**B**) Root cell membrane integrity. Bars correspond to 500 nm. The red arrows in the NaCl-treated figures represent irregular cells and cell membrane structures. The red arrows in the control and NaCl + 200 mg/L PC-treated figures indicate intact cells and cell membrane structures. Measurements were performed using the root tips of peach plants. The data were sampled at 11:00 a.m. on the 11th day after the salt stress treatment.

The integrity of the cell structure and membrane is crucial in the process of plant tolerance against abiotic stress. Therefore, we used transmission electron microscopy to observe the cell structure and cell membrane integrity of peach tree roots. Our observations revealed that, in contrast to roots in the control, the cell structure of the roots under the NaCl treatment were severely disrupted (Figure 4A). The integrity of root cells in the control group was the best, and the structural integrity of root cells under NaCl treatment was the worst. The root cells of the three treatments with PC added had higher cell integrity than those treated with NaCl. Among them, the cells treated with NaCl + 200 mg/L PC had the highest integrity. It can be seen from Figure 4B that the root cell membrane structure under NaCl treatment has been severely damaged, and the cell fluid leaks, resulting in severe damage to cell function. The addition of PC salt treatment can protect the cell membrane structure. Among them, the addition of 200 mg/L and 400 mg/L of PC maintained cell membrane integrity under the applied salt stress conditions.

### 2.5. PC and PA Content, and PLD Activity in the Leaves and Roots under Salt Stress

**Figure 5:**
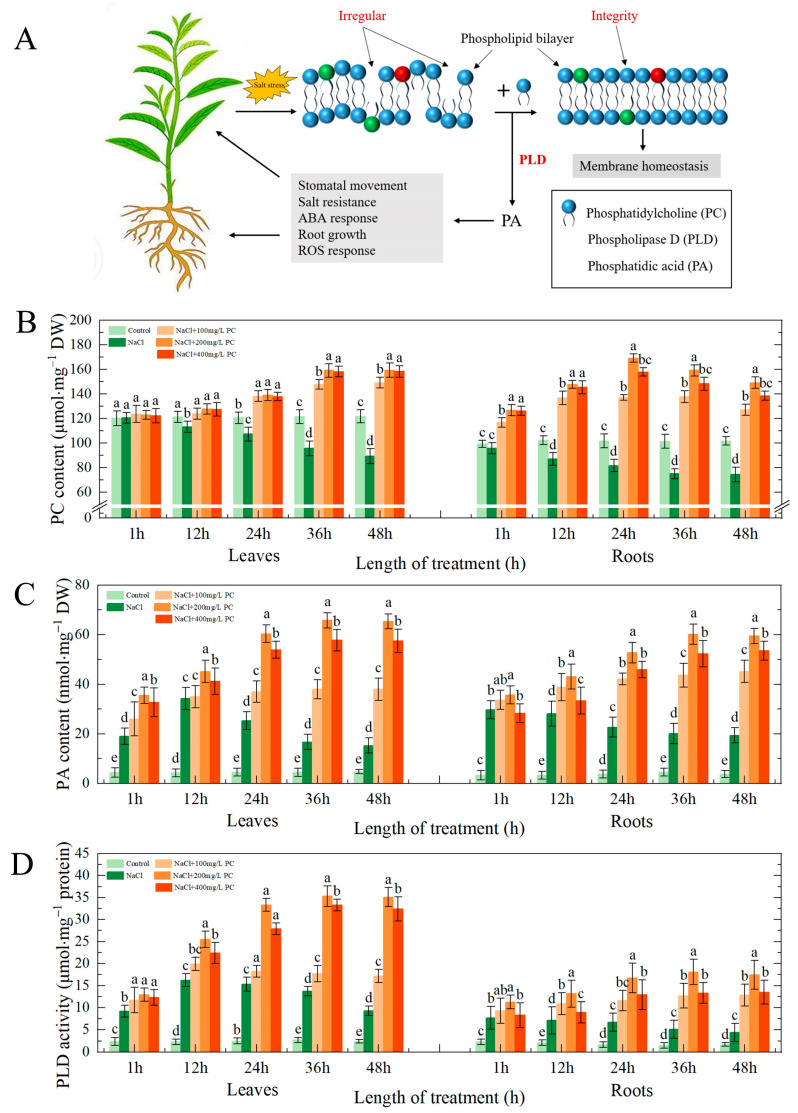
Cell membrane structure, PC, PA content, and PLD activity. (**A**) Effect of salt stress on the phospholipid bilayer of the plant cell membrane; (**B**) PC content; (**C**) PLD activity; (**D**) PA content. The error bar represents the standard deviation of the mean (*n* = 3). Different lowercase letters indicate significant differences among treatments (Duncan test, *p* < 0.05).

The plant cell membrane is the first that is attacked under salt stress exposure. PC is a phospholipid with the highest content in the phospholipid bilayer of the cell membrane. Therefore, the content of PC in the cell membrane is essential for plant tolerance to salt stress (Figure 5A).

We found that under salt stress, the leaves and roots of peach seedlings could absorb exogenous PC (Figure 5B), and the activity of PLD in the cell was significantly enhanced (Figure 5D). Of the examined treatment, NaCl + 200 mg/L PC had the most pronounced impact on the PLD activity in the leaves and roots. PLD decomposes PC, producing PA. As can be seen in Figure 5C, the content of PA, which is a secondary messenger, was significantly increased. PA plays also an important role in the tolerance of plants to salt stress.

### 2.6. Exogenous Application of PC Enhances Root Cell Activity

**Figure 6:**
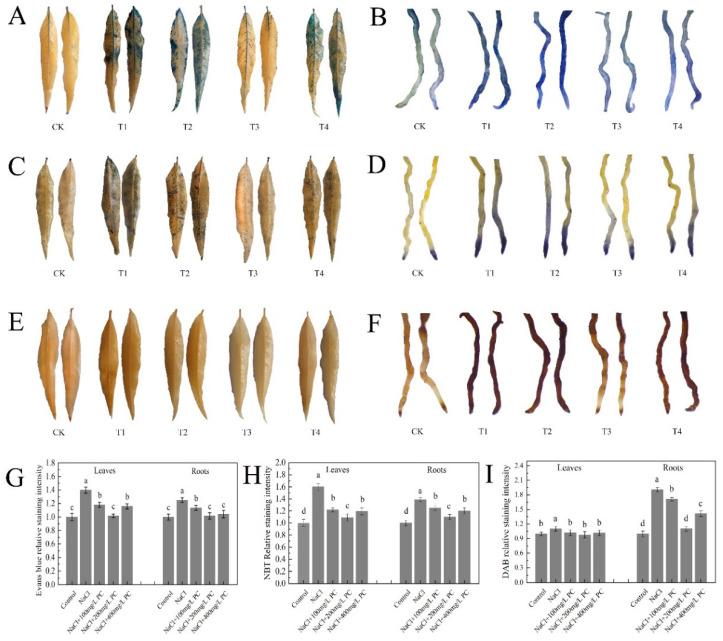
Leaves and roots staining. (**A**) Evans blue-staining of leaves; (**B**) Evans blue-staining of roots; (**C**) NBT staining of leaves; (**D**) NBT staining of roots; (**E**) DAB staining of leaves; (**F**) DAB staining of roots; (**G**) Relative Evans blue-staining intensity; (**H**) Relative NBT staining intensity; (**I**) DAB relative staining intensity. Control treatment (CK, a negative control group); tree treatment with only 70 mmol/L NaCl (T1); tree treatment with 70 mmol/L NaCl and 100 mg/L PC (T2); tree treatment with 70 mmol/L NaCl and 200 mg/L PC (T3); tree treatment with 70 mmol/L NaCl and 400 mg/L PC (T4). The data were sampled at 8:00 a.m. on the 12th day after the salt stress treatment. Measurements were performed using the root tips of peach plants. Error bar represents standard deviation of the mean (*n* = 3). Different lowercase letters indicate significant differences among different treatments (Duncan test, *p* < 0.05).

Evans blue-stained leaves and roots under salt stress are displayed in Figure 6A,B. The application of PC increased the activity of the leaf and root cells. The relative Svensland staining intensity visible in Figure 6G shows that the highest leaf cell activity was observed at the PC concentration of 200 mg/L; the root cell activity was the highest at the PC concentrations of 200 mg/L and 400 mg/L. 

SOD is present in animals and plants. Its function is to scavenge superoxide anion free radicals. NBT staining is widely used for the determination of SOD activity due to its simple operation and high-sensitivity [19]. As illustrated in Figure 6C,D, the relative staining intensity of the leaves and roots of the plants treated with exogenous PC was lower than that of the only salt stress-treated plants. The relative staining intensity of NaCl + 200 mg/L PC was the lowest, and the relative staining intensity of leaves is 31.88% less than NaCl treatment. The relative staining intensity of roots was lower by 20.63% compared with NaCl treatment (Figure 6H).

POD decomposes hydrogen peroxide (H_2_O_2_) to produce water and release oxygen. DAB staining is used to detect the active peroxidase sites in cells. As shown in Figure 6E, the difference in DAB staining of peach tree leaves was small. As illustrated in Figure 6F, the DAB staining of roots of the studied peach trees produced significantly different results. The relative staining intensity of the 200 mg/L PC treatment was the lowest, which was 42.10% lower than that of the NaCl treatment (Figure 6I). 

### 2.7. Electrolyte Leakage Rate and SOD, POD, MDA, Proline, and Soluble Sugar Contents

**Figure 7:**
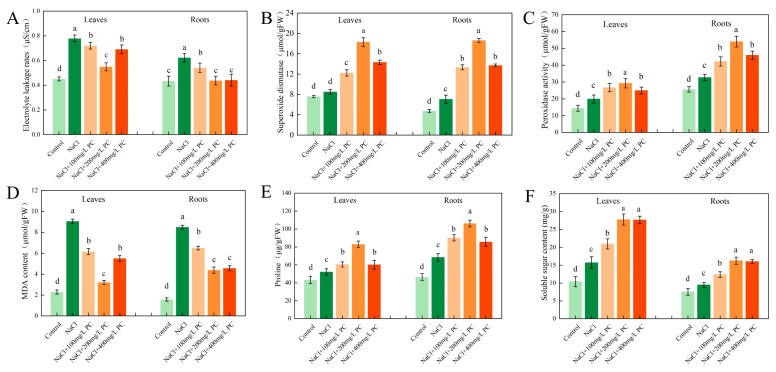
Antioxidant enzyme activity and osmotic balance in leaves and roots. (**A**) Electrolyte extravasation rate; (**B**) Superoxide dismutase; (**C**) Peroxidase activity; (**D**) Malondialdehyde content; (**E**) Proline content; (**F**) Soluble sugar content. The error bar represents the standard deviation of the mean (*n* = 3). Measurements were performed using the root tips of peach plants. The data were sampled at 9:00 a.m. on the 12th day after the salt stress treatment. Different lowercase letters indicate significant differences among different treatments (Duncan test, *p* < 0.05).

Notably, salt stress exposure increased the cell electrolyte leakage rate, whereas the PC treatment reduced the leaf and root electrolyte leakage rate (Figure 7A). Therefore, the application of PC can protect plant cells from the adverse effects of salt stress and attenuate their damage. As visible in Figure 7B, the SOD activity of 200 mg/L PC was the highest. The SOD activity of the leaves in that treatment was 115.82% higher than that of the control; the SOD activity of the roots was 164.06% higher than that of the control. The results of the NBT staining and SOD showed that exogenous application of PC under salt stress can remove the superoxide anions accumulated in the plant, protecting plant cells and reducing damage.

As can be seen in Figure 7C, compared with 100 mg/L PC and 400 mg/L PC, exogenous application of 200 mg/L PC had the most significant increase in POD activity in leaves and roots. The POD activity of the leaves in that treatment was 47.47% higher than that of the NaCl treatment; the POD activity of the roots was 66.05% higher than that of the NaCl treatment. Its effect is beneficial as H_2_O_2_ is released in the cell, which reduces the damage to plants by POD. The high amount of MAD in plants indicates a severe degree of plant cell membrane damage as it is a manifestation of the degree of peroxidation. As can be observed in Figure 7D, the content of MDA in the salt stress treatment was higher than that in the control group, indicating that salt stress had increased the degree of peroxidation in the leaf and root cell membranes. However, the addition of PC significantly reduced the peroxidation degree of the plant cell membrane. In the NaCl + 200 mg/L PC treatment, the MDA contents in the leaves was 64.53% lower, respectively, than those in the NaCl treatment. The 200 mg/L PC and 400 mg/L PC treatments could significantly reduce MDA content in roots, which were 48.41% and 46.29% lower than NaCl treatments, respectively. 

To adapt to adverse conditions, such as salt stress, plants actively accumulate proline and soluble sugars, reduce their osmotic potential, and promote water absorption by the root system to adapt to the external environmental changes. The accumulation of another important osmotic adjustment substance in the vacuole, proline, also plays a role in regulating the cytoplasmic osmotic balance. As illustrated in Figure 7E, under the salt stress treatments of our experiment, the contents of proline in the plant increased. The plants in the NaCl + 200 mg/L PC treatment accumulated more proline and soluble sugars than those in the treatment with NaCl. NaCl + 200 mg/L PC treatment had the most significant accumulation of proline in leaves and roots, which were 59.05% and 54.93% higher than NaCl treatment, respectively. As illustrated in Figure 7F, NaCl + 200 mg/L PC treatment and NaCl + 400 mg/L PC treatment were the most significant for soluble sugar accumulation in leaves and roots. The soluble sugar contents in leaves and roots of NaCl + 200 mg/L PC treatment and NaCl + 400 mg/L PC treatment were 76.46% and 75.76% higher, 71.59% and 69.27% higher than those of NaCl treatment, respectively. This accumulation had a beneficial effect on the plants as it adjusted plant osmotic potential in response to the salt stress conditions.

## 3. Discussion

### 3.1. PC, PA Contents, and PLD Activity in the Cells Exposed to Salt Stress

High salinity is commonly due to high-concentrations of Na^+^ and Cl^−^ in the soil solution, resulting in hyperosmotic and hyperionic conditions that impede soil water and nutrient absorption by plants [20]. Plant cell membrane controls the entry and exit of most ions and macromolecular substances. It is the interface between plants and the outside environment that serves for information and material exchange; it is also the first barrier used by plants to resist external damage [21]. The cell membrane is a viable organelle, which can adjust the membrane structure in different states by changing its chemical structure or molecular morphology [5]. The membrane system is a plant part that is sensitive to injury. PC is an important component of membrane lipids. The steady state of its content determines the normal biological function of membranes [22]. Plant salt tolerance is closely related to the structure and function of cell membranes, and the lipid content and composition of plant cell membranes under salt stress had undergone considerable changes [23,24]. Our research revealed that PC not only is the main component of the structural basis of biological membranes, but its metabolites are also used as signal substances involved in the growth and development of plants and their response to salt stress. 

PA is an important phospholipid messenger that has been recently discovered [14]. It is involved in a variety of adversity responses and hormone information transmission processes. In contrast to the high levels of structural phospholipids in the eukaryotic cell, PA content is low, accounting for only 1–2% of the total phospholipids, in the cell [13,25]. Previous studies found that PA is involved not only in various cellular processes, such as cytoskeleton rearrangement, vesicle transport, and membrane lipid biosynthesis [26,27], but it is also a regulator that participates in the physiological response of plants to a variety of biotic and abiotic stresses. These adverse abiotic conditions include low-temperature, freezing, dehydration, drought, salt stress, nutrient deficiency, and mechanical damage [28,29,30]. Some scholars believe that the functions of PA messenger and classic second messenger (Ca^2+^ and cAMP) are equally important [31]. Here, we established that the exogenous application of PC under salt stress promotes PLD activity. In turn, this enhanced activity leads to the decomposition of intracellular phospholipids and the production of PA, thereby regulating and improving the tolerance of the peach seedlings to the exposure to salt stress.

### 3.2. Effect of the Exogenous Application of PC on the Photosystem under Salt Stress Exposure

Under salt stress, plant chloroplasts are destroyed, and the related photosynthetic enzyme activities decrease, resulting in a decline in the photosynthetic rate of plants [3]. Previous studies have found that phospholipids are essential for the photosynthesis in higher plants and cyanobacteria. The content of phospholipids in plant cell membranes changes in response to stress to retard or reduce the damage [32,33]. The chloroplast in the leaves is composed of three parts: envelope, matrix, and thylakoid, which is the main location in plants where photosynthesis occurs [34]. Kim found that phosphatidylcholine is required for the efficient formation of photosynthetic membrane and B800-850 light-harvesting complex in rhodobacter sphaeroides [35]. It can be seen from Figure 1D–F that the exogenous application of PC under salt stress can protect the contents of chlorophyll a, chlorophyll b, and carotenoids in leaves. Our research found that the application of PC under salt stress can stabilize the phospholipid content in leaf cells, ensure the normal function of cells, and facilitating the maintenance of normal photosynthesis under salt stress. Compared with breeding and biotechnological methods to alleviate salt stress damage, the application of PC is simple and convenient, and can save a lot of human and financial resources.

Stomata are the channels through which higher plants exchange water and gas with the outside environment. More than 90% of the water loss from plants is via leaf stomatal transpiration. Investigations of the mechanism of stomatal movement are of substantial significance for the in-depth understanding of plant stress adaptation, water use, and signal transduction mechanisms. Jiang et al. evidenced that the abscisic acid (ABA)-induced stomatal closure process in Arabidopsis required the participation of PA in the regulation of microtubule depolymerization [36]. Furthermore, Zhang et al. found that PLD-derived PA was essential in microtubule tissue process regulation under salt stress; PA and MAP65-1 interacted to regulate the microtubule structural organization and salt tolerance [37]. Our present study revealed that under salt stress, PA, which is a signal molecule produced by PC decomposition, is critically involved in the ABA-induced stomatal opening and closing. The exogenous application of PC under salt stress can thus regulate the opening of stomata and ensure that cells can normally communicate with the outside world. This can also explain the normal entry of CO_2_ into the mesophyll by opening the stomata, enabling the maintenance of normal photosynthesis in the leaves.

### 3.3. Effects of PC on the Root Structure, Growth, Cell Components, and Cell Membrane Integrity under Salt Stress

In plants, PLD and PA are considered to be involved in the promotion of cell elongation of pollen tubes and root hairs [38,39]. Additionally, they participate in the adaptation processes associated with plant growth under phosphorus deficiency and high osmotic stress [28,40]. PLD activation was found to occur under high permeability conditions [31,41]. Moreover, Hong et al. established that PLD can also promote plant growth under high-salinity and high-osmotic stress caused by the lack of water [42]. Under these adverse conditions, PLD can enhance the plant nitrogen absorption and increase the root surface area, promoting greater water absorption and utilization by plants. The lipid profile analysis of root tissues in a previous study indicated that PA molecules may participate in sensing nutrition and osmotic signals to regulate root growth. 

In agricultural practice, grafting has been described to increase salt tolerance by excluding or restricting toxicion accumulation in shoots [43]. Previous research found that the influence of rootstock on a scion’s salt and water stress tolerance is due to: using a larger and vigorous root systems capable of absorbing water and nutrients much more efficiently [44]. However, the selection of rootstocks is limited, and it is difficult to concentrate multiple excellent traits on one rootstock. Therefore, the selection of rootstocks to resist salt stress not only consumes a lot of time and money, but also it is difficult to retain the original excellent characters of the species [45]. Our present research evidenced that under salt stress, the exogenous application of PC enhanced the activity of PLD and increased the content of PA in the cells. Notably, the exogenous application of PC increased the root growth of the treated peach seedlings as compared with that in the control, especially the number of fine roots (Figure 3B). Using transmission electron microscope observations of the root cells, we found that the exogenous administration of PC had a positive effect on maintaining cell integrity and cell-membrane structure. Bewley (1979) showed that plant drought tolerance depends on the ability to limit membrane damage during water stress [46]. It can be seen from Figure 4 that the absorption of PC by peach can stabilize the cell membrane structure and enhance the integrity of the cell membrane, thereby enhancing the tolerance to salt stress. The potential underlying mechanism of action could be through the maintenance of the cell membrane phospholipid homeostasis by PC and the enhanced salt tolerance of the cell membrane structure via PA regulation. Therefore, the application of PC to alleviate salt stress damage can save the financial resources and time of rootstock selection, and the operation is simple and the excellent characters of the species are retained to a greater extent.

### 3.4. Application of PC on Maintaining Cell Activity under Salt Stress

Leaves are the main photosynthetic organs in higher plants. The chloroplast in the leaf consists of three parts: envelope, stroma, and thylakoid, which is the main place for photosynthesis of plants [47]. In trees and other perennial woody species, the role of the root system is even more important than that in small annual plants [48]. Therefore, the activity of plant leaf and root cells under salt stress determines whether plants can maintain normal growth and development. By staining the leaves and roots with Evans blue, we found that the application of 200 mg/L PC under salt stress can protect the physiological activity of leaf and root cells to the greatest extent, significantly reducing the electrolyte extravasation rate, which is important for protecting the integrity of cell membranes and the maintenance of normal cell function.

Under salt stress, plants adjust accordingly their physiological and biochemical processes to adapt, including ion and osmotic homeostasis regulation, as well as the control and repair (detoxification) of stress-induced damage [49]. SOD is an antioxidant metal enzyme that catalyzes the dismutation of superoxide anion radicals to generate oxygen and H_2_O_2_. It plays an important role in plant tolerance against biotic stress. Under adverse conditions or senescence, H_2_O_2_ accumulates in the plant due to increased active oxygen metabolism. H_2_O_2_ can directly or indirectly oxidize nucleic acids, proteins, and other biological macromolecules in the cell and damage the cell membrane, thereby accelerating plant senescence and the degradation of cells. POD is another antioxidant enzyme located in the peroxisome of the cell, which plays an important role in preventing the damage by oxygen metabolites [50]. It can be seen from Figure 6 and Figure 7 that the exogenous application of 200 mg/L PC under salt stress increased the SOD activity of the leaves and roots, and the activity of root POD. We speculate that PA produced by PC can activate the activities of SOD and POD. It exerted a positive effect in plants by removal of superoxide anions and peroxide.

### 3.5. Effect of PC Application on MDA, Proline, and Soluble Sugar Contents

Plant organs are damaged under salt stress, and membrane lipid peroxidation often occurs. MDA is the final decomposition product of membrane lipid peroxidation, and its content can reflect the degree of damage to plants [51]. Another important aspect of salt tolerance mechanisms is the capacity of plant cells to adapt osmotically by the plant tissue accumulation of organic solutes, such as free proline, endogenous glycine betaine, soluble sugar, and protein. Hence, their increased contents might indicate the degree of tolerance to osmoregulation-induced stress [52]. These osmotic solutes maintain osmotic balance and normal turgor and control water influx (reduce the efflux). It can be seen from Figure 7 that exogenous application of PC under salt stress reduced the MDA content and the cell membrane damage in the experimental plants. The content of proline and soluble sugar increased significantly. PLD-produced PA increases rapidly in cell-suspension cultures of tomato and alfalfa subject to salt stress and in the dehydrated leaves of the resurrection plant Craterostigma plantagineum [53]. We speculate that PLD decomposes PC to generate organic solutes and maintain the osmotic balance of intracellular organic solutes and part of the PA is produced by PLD to regulate the salt tolerance of plants.

## 4. Materials and Methods

### 4.1. Plant Material and Exogenous PC Treatment

Experiments were performed in the experiment center of Shandong Agricultural University (Tai’an, China) in June 2020. The 101-year-old ‘lu xing’ peach, *Prunus persica* (L.) Batsch., seedlings were planted in 2.5 L soil pots. These pots were then placed in a glass greenhouse with a day/night temperature regime of 26 °C/18 °C, a natural photoperiod of around 12.5 h, and constant relative humidity of 30%.

The experiment set up included five treatments, and each treatment was repeated in 20 pots (replicates). In the preliminary salt stress screening test, we found that salt-induced damage to the peach seedlings began to appear after their treatment with 70 mmol/L NaCl for 10 days, which met the test requirements. Therefore, in this experiment, we chose to use 70 mmol/L NaCl as the salt stress treatment concentration. We used the following treatments when the plants grew to a stage of having from 10 to 12 fully expanded leaves: control treatment (CK, a negative control group); tree treatment with only 70 mmol/L NaCl (T1); tree treatment with 70 mmol/L NaCl and 100 mg/L PC (T2); tree treatment with 70 mmol/L NaCl and 200 mg/L PC (T3); tree treatment with 70 mmol/L NaCl and 400 mg/L PC (T4). In this experiment, PC and NaCl were directly applied to the soil at a reagent dosage of 100 mL per tree. All PC treatments were applied in soil pots at 5 p.m. to avoid excessively high-temperatures.

### 4.2. PC and PA Content and PLD Enzyme Activity in Plants

Nine leaves and six roots were randomly selected from each treatment, ground under liquid nitrogen and used for later use. To determine the amounts of plant PC in the samples, cover microtiter plate wells with purified plant PC antibody, produce solid-phase antibody, and then add PC to wells. After thorough washing, a combined Horseradish Peroxidase (HRP)-labeled PC antibody was used as an antibody-antigen-enzyme-antibody complex. Upon addition of 3,3′,5,5′-Tetramethylbenzidine (TMB) substrate solution, the TMB substrate turned blue. The reaction catalyzed by the HRP enzyme was terminated by the addition of sulphuric-acid solution, leading to a color change that was detected spectrophotometrically at 450 nm. We measured the concentration of plant PC/PA in the samples by comparing the OD of the samples to the standard curve. To compute the sample concentration, we substituted the sample OD value into the equation.

A PA level dynamic assay was performed as described previously [54].

The PLD activity was then determined following the method previously described by Wang [55]. PLD catalyzes the hydrolysis of the phosphatidyl diester bond at the end of PC to produce PA and choline. Choline is next catalyzed by choline oxidase to generate betaine and hydrogen peroxide. Further, 4-aminoantipyrine and double-distilled phenol were oxidized to a pink substance with a characteristic absorption peak at 500 nm. W is the sample mass of 0.1 g, PLD enzyme activity (nmol/min/g) = 16.7 × A measuring tube/A standard tube/W.

### 4.3. Measurements of the Net Photosynthetic Rate and SPAD Values

On the first day of our experiment and every two days following the salt stress treatment (until the ninth day), we assessed the net photosynthetic rate and chlorophyll content of peach leaves. We measured the leaf net photosynthetic rate in the leaves of peach trees from the treatments using a CIRAS-3 Portable Photosynthesis System (PP Systems, Amesbury, MA, USA). All leaves were equilibrated at a constant flow rate of 500 mL min^−1^ and a CO_2_ concentration of approximately 400 μmol mol^−1^ at PAR of 1000 μmol M^−2^ S^−1^. Between 9:30 and 11:00 a.m., a SPAD-502 PLUS chlorophyll meter was used to measure the amount of chlorophyll in fully expanded leaves (Spectrum Technologies, Aurora, IL, USA). On the ninth day, we used a previously reported procedure to determine the content of chlorophyll a, chlorophyll b, and carotenoids [56].

### 4.4. Determination of Stomatal Area, Root Growth, and Root Cell Integrity

Three pots of peach seedlings were randomly selected for each treatment, and three leaves were selected from each peach seedling, with a total of nine leaves for each treatment. Nine leaves of the same developmental stage were chosen on the tenth-day post-salt stress treatment to measure the size and density of the stomata. To perform the measurements, we stained the leaf epidermis with clear acrylic nail polish. The nail polish was taken off with forceps once it had dried. The solid polish was then put on a microscope slide and examined with a fluorescence microscope at 400× magnification (AXI0, Carl Zeiss, Jena, Germany). We make nine slides per treatment. We randomly selected three 3.2-mm^2^ areas on each slide and took a picture. We used ImageJ version 1.48 to count the number of stomata and quantify their length and width (National Institutes of Health, Bethesda, MD, USA). 

Next, we randomly collected six roots of fresh peach seedlings, rinsed them with water, and determined the root configuration parameters using the professional version of WinRHIZO Root Analysis System (Epson V 700).

Transmission electron microscopy was further employed to examine the plant root cells’ integrity, as well as the membrane structure of the cells. Samples were then embedded in Epon812 epoxy resin before incubation and three sequential curing phases at 37 °C, 45 °C, and 65 °C, each for 24 h. We sectioned samples and used lead uranyl acetate to stain them. Finally, we put samples in the machine for testing and capture images.

### 4.5. Measurements of Root Cell Activity, NBT and DAB Staining, and Electrolyte Leakage Rate

Nine root tips from each treatment were selected for staining. Evans blue staining examination has been confirmed as a reliable method for microscopic cell death determination. We used that method to determine cell death, as previously described by Baker and Mock [57], with slight changes. Root samples were vacuum infiltrated in 0.1 % Evans blue (*w*/*v*) solution for 5 min before staining at room-temperature for 3 to 5 h. The samples were then rinsed in phosphate-buffered saline (PBS) containing 0.05% (*v*/*v*) Tween-20. ImageJ was utilized to evaluate the relative staining intensity after an image was taken using an electron microscope. A darker root image indicated a lower cell activity.

Further, we prepared 0.5 mg/mL of nitroblue tetrazolium chloride (NBT) staining solution, poured the staining solution into an Erlenmeyer flask, added leaves and roots, and inserted a vacuum pump. The samples were incubated in the dark at 28 °C for 8 h. Next, we discarded the staining solution, and boil in ethanol for 5 min: lactic acid: glycerol (6 mL:2 mL:2 mL) fixing solution until all chlorophyll is removed, cooled, and 10 mL of absolute alcohol is added. Finally, we perform observation staining.

We prepared 1 mg/mL of diaminobenzidine (DAB) staining solution and adjusted its pH to 5.8 with NaOH. Then, we transferred the staining solution into an Erlenmeyer flask, added pretreated plant tissues, and inserted a vacuum pump. We next incubated the mixture in the dark for 8 h at 28 °C. Further, we discarded the staining solution and boiled the sample in a fixative solution containing ethanol: lactic acid: glycerol (6 mL:2 mL:2 mL) fixative solution for 5 min until complete removal of the chlorophyll was performed. Then, we cooled the samples, added absolute alcohol 10 mL, and observed the staining.

A simple approach was implemented to determine the electrolyte leakage rate (MII), as reported earlier [58]. Three pots of peach seedlings were randomly selected from each treatment, and three leaves and three roots were randomly selected from each treatment. Peach tree leaves and roots were cleansed thoroughly, and little discs of root tissue were punched out. The initial electrical conductivity (initial EC) of the solution was measured using a DDS-307 conductivity meter after this immersion (Shanghai Precision Scientific Instrument Co., Ltd., Shanghai, China). After heating the samples at 100 °C for 20 min, we utilized the same apparatus to determine the final EC of the solution. The following formula was used to calculate MII: MII (%) = (initial EC/final EC) × 100.

### 4.6. Determination of the Antioxidant system

Measurements were performed using the leaves and root tips of peach plants. Three pots are required for each treatment. The data were sampled at 9:00 a.m. on the 12th day after the salt stress treatment. Superoxide dismutase (SOD) and peroxidase (POD) activities were measured following the protocol reported previously by Liu [59]. Malondialdehyde (MDA) and proline contents were measured as described by Tang [60]. We conducted the determination of soluble sugar content as described by Zhao [56].

### 4.7. Statistical Analysis

We collected three biological replicates for each treatment. Origin version 9.4 was used to conduct all statistical analyses. Duncan multiple range tests, which are included in SPSS version 20.0, were performed to detect any statistically significant differences in the mean values (IBM SPSS, Chicago, IL, USA). The threshold of statistical significance used for all tests was *p* < 0.05.

## 5. Conclusions

In this study, the growth and development of peach seedlings were severely negatively affected by salt stress. The photosynthetic system of the seedlings failed to function normally, the root development was blocked, root cell morphology was abnormal, and the cell membranes were damaged. The exogenous application of PC alleviated the toxic effects of salt stress; the most significant positive effect was observed at a PC concentration of 200 mg/L. We can make the following speculations. First, the exogenous PC absorbed by plants under salt stress protects the homeostasis of cell membrane phospholipids, ensuring normal plant growth and development. Second, PLD can decompose the remaining PC to produce PA, which acts as a secondary messenger to regulate plant tolerance against salt stress. However, the regulation of PA levels in the plant under salt stress conditions needs further research.

## Figures and Tables

**Table 1:** Stomatal length, width, and area.

	*Prunus persica* (L.) Batsch. Stomatal Measurements		
	Length (μm)	Width (μm)	Area (μm^2^)
Control	22.8 ± 3.7 a	15.4 ± 1.5 a	329.1 ± 0.51 a
NaCl	21.8 ± 2.1 b	11.3 ± 1.3 d	213.5 ± 0.21 d
NaCl + 100 mg/L PC	21.3 ± 2.5 b	12.8 ± 1.2 c	245.3 ± 0.20 c
NaCl + 200 mg/L PC	21.7 ± 3.5 b	14.5 ± 1.8 b	293.3 ± 0.61 b
NaCl + 400 mg/L PC	21.5 ± 2.3 b	13.1 ± 1.6 c	267.9 ± 1.01 c

Note: Mean ± standard deviation (*n* = 3). The different letters indicate significant differences at a level of *p* < 0.05; the same below.

**Table 2:** Peach tree root length, surface area, root volume, tips, and forks.

	Length (cm)	Surface Area (cm^2^)	Root Volume (cm^3^)	Tips	Forks
Control	1506.6 ± 92.7 a	251.6 ± 17.1 a	3.3 ± 0.2 a	2927 ± 112 a	17602 ± 991 a
NaCl	793.7 ± 64.3 e	131.1 ± 9.9 e	1.7 ± 0.1 d	1457 ± 125 e	5628 ± 481 d
NaCl + 100 mg/L PC	907.7 ± 66.9 d	160.4 ± 8.7 d	2.3 ± 0.1 c	1658 ± 97 d	7991 ± 698 c
NaCl + 200 mg/L PC	1311.5 ± 97.2 b	184.9 ± 8.3 b	2.8 ± 0.2 b	2433 ± 131 b	10361 ± 852 b
NaCl + 400 mg/L PC	1029.2 ± 95.5 c	170.9 ± 7.8 c	2.3 ± 0.2 c	2202 ± 148 c	7952 ± 650 c

Note: Mean ± standard deviation (*n* = 3). The different letters indicate significant differences at a level of *p* < 0.05.

## Data Availability

Not applicable.
